# The Transumbilical Laparoendoscopic Single-Site Extraperitoneal Approach for Pelvic and Para-Aortic Lymphadenectomy: A Technique Note and Feasibility Study

**DOI:** 10.3389/fsurg.2022.863078

**Published:** 2022-04-15

**Authors:** Shiyi Peng, Ying Zheng, Fan Yang, Kana Wang, Sijing Chen, Yawen Wang

**Affiliations:** ^1^Department of Gynecology, West China Second Hospital, Sichuan University, Chengdu, China; ^2^Key Laboratory of Obstetrics, Gynecologic and Pediatric Diseases and Birth Defects of Ministry of Education, West China Second Hospital, Sichuan University, Chengdu, China; ^3^Department of Obstetrics and Gynecology, Shanxi Bethune Hospital, Taiyuan, China

**Keywords:** extraperitoneal approach, pelvic lymphadenectomy, para-aortic lymphadenectomy, laparoendoscopic single-site (LESS) surgery, ovarian cancer, endometrial cancer

## Abstract

**Background:**

Nowadays, lymphadenectomy could be performed by the transperitoneal or extraperitoneal approach. Nevertheless, each approach has its own advantages and disadvantages. Under these circumstances, we developed a transumbilical laparoendoscopic single-site (TU-LESS) extraperitoneal approach for lymphadenectomy. In this research, the primary goal is to demonstrate the feasibility of the novel approach in systematic lymphadenectomy and present the surgical process step-by-step.

**Methods:**

Between May 2020 and June 2021, patients who had the indications of systematic lymphadenectomy underwent lymphadenectomy via the TU-LESS extraperitoneal approach. This new approach was described in detail, and the clinical characteristics and surgical outcomes were collected and analyzed.

**Results:**

Eight patients with gynecological carcinoma were included in the research, including four with high-risk endometrial cancer and four with early-stage ovarian cancer. The TU-LESS extraperitoneal approach for pelvic and para-aortic lymphadenectomy was successfully performed in all patients without conversion. In all, a median of 26.5 pelvic lymph nodes (range 18–35) and 18.0 para-aortic lymph nodes (range 7–43) were retrieved. There was a median of 166.5 min of surgical time (range 123–205). Patients had speedy recoveries without complications. All patients had positive pain responses after surgery, as well as satisfactory cosmetic and body image outcomes.

**Conclusion:**

Our initial experience showed that it is feasible to perform systematic lymphadenectomy with the TU-LESS extraperitoneal approach. And this new approach may provide a new measure or a beneficial supplement for lymphadenectomy in gynecologic cancer.

## Introduction

**Figure 1:**
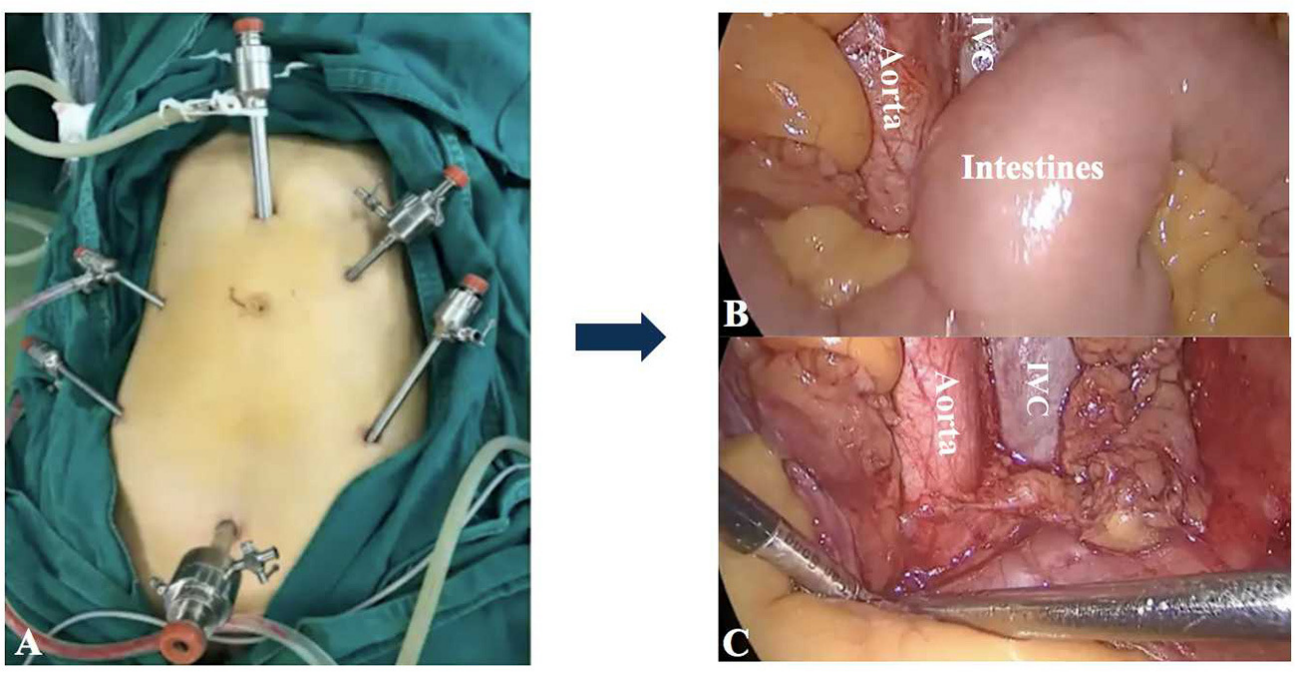
The transperitoneal approach for PALN. **(A)** The placement of trocars. It was difficult to achieve adequate exposure of para-aortic regions for PALN because of the interference of intestines **(B,C)**. IVC: Inferior vena cava.

Lymphadenectomy is paramount for precise staging and tailoring treatment of gynecological malignancies. Compared to laparotomy, laparoscopic surgery caused less surgical trauma and fewer wound complications. The feasibility and safety of minimally invasive surgery for lymphadenectomy has been well-investigated and proved (1, 2). Currently, laparoscopic lymphadenectomy is performed either trans- or extraperitoneally. Dissection of the pelvic lymph nodes (LNs) is easier with the transperitoneal approach; however, the intestinal disruption is a major barrier for para-aortic lymphadenectomy (PALN) (Figure 1). The extraperitoneal approach has been described as a solution to resolve this problem. Without the interference of bowels, the extraperitoneal approach provides an easier access to the infrarenal para-aortic LNs with lower risk of intestinal and urinary injuries (3). The full exposure of surgical field achieved a higher para-aortic LN yield compared to the transperitoneal route (4, 5).

**Figure 2:**
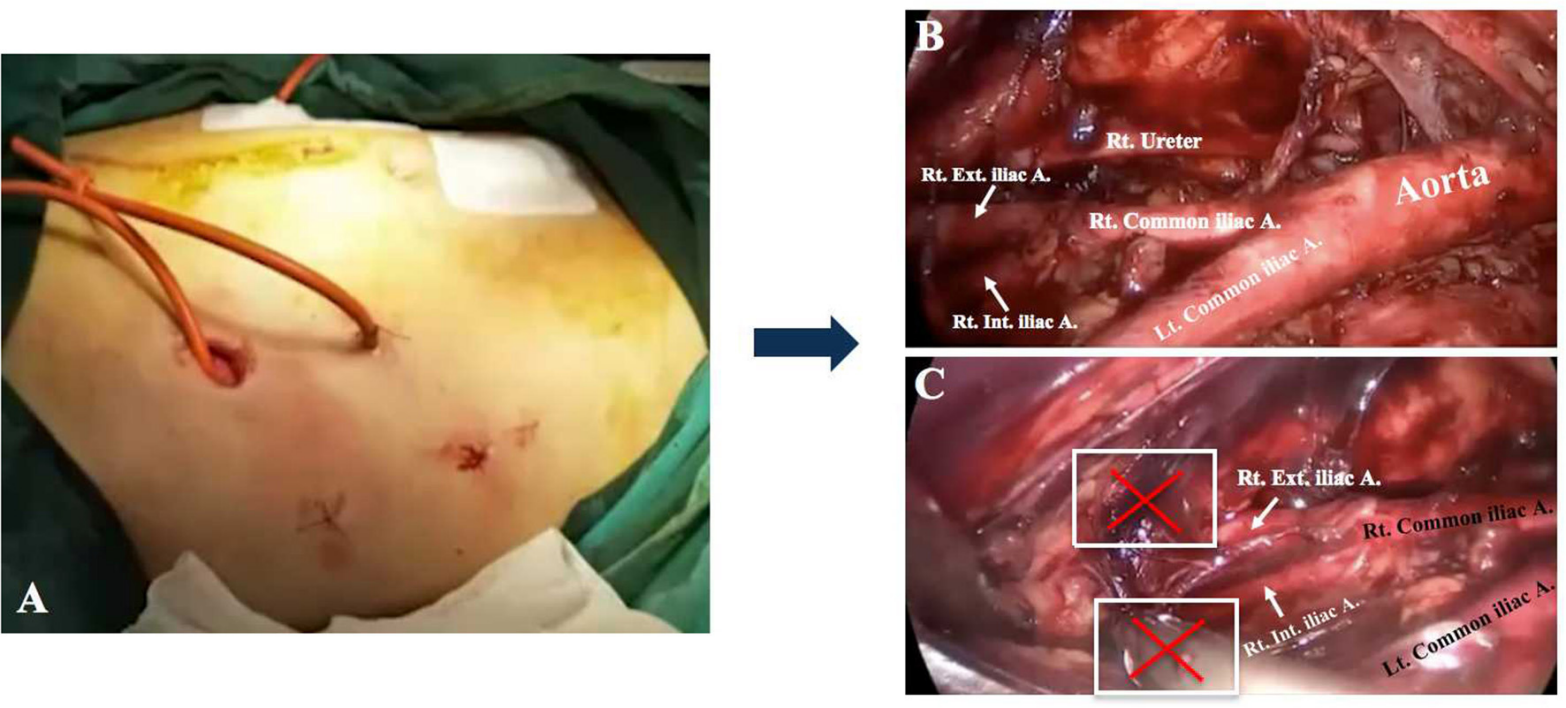
The lateral extraperitoneal approach for lymphadenectomy. **(A)** The lateral incisions. **(B)** Para-aortic LN dissection. Bilateral obturator fossae were challenging to reach when performing pelvic lymphadenectomy **(C)**.

Laparoendoscopic single-site (LESS) surgery has emerged as a minimal invasive surgical approach, which could further minimize the surgical trauma compared to multi-port laparoscopy surgery (6). LESS is as safe and effective as the traditional laparoscopy in the gynecologic surgery (7). Compared to patients in the multi-port laparoscopy group, patients in the single-port laparoscopy group attained mild pain with less analgesic consumption and shorter hospital stay (8–11). The single-port left iliac extraperitoneal PALN was first described by Guoy et al. (12). Subsequently, Lambaudie et al. (13) and Beytout et al. (14) introduced similar single-port lateral approaches. These results indicated that the number of para-aortic LNs retrieved by the single-port lateral extraperitoneal approach was compatible with that of the multi-port extraperitoneal route (3, 10, 13). In spite of this, the most common lateral extraperitoneal technique restraints access to the obturator fossa which impedes pelvic lymphadenectomy (PLN) (15) (Figure 2). Under these circumstances, PLN and other staging procedures sometimes need extra incisions, which increases the amount of trauma experienced throughout the operation. Thus, the TU-LESS extraperitoneal approach, which combines the strengths of LESS with that of extraperitoneal approach was developed to achieve PLN and PALN in a minimal invasive way. This study aims to describe the details of surgical procedures and present our preliminary experience with the TU-LESS extraperitoneal approach for PLN and PALN in order to further evaluate its feasibility.

## Methods

### Patients

This study included eight patients from May 2020 to June 2021. Patients who had indications of systematic lymphadenectomy and were candidates for LESS surgery were eligible for inclusion. Clinical data were collected, including demographics, pathological features, and perioperative outcomes of patients who had the surgery. The study was approved by the institutional review board of the West China Second Hospital, Sichuan University, and all participants provided their written informed consent to participate in this study. The duration of lymphadenectomy time was defined as the interval from the first incision of skin to completion of lymphadenectomy, excluding subsequent procedures such as hysterectomy. The failure of the TU-LESS extraperitoneal approach was defined as the conversion to a transperitoneal approach *via* laparoscopy or open surgery; and intraoperative complications included peritoneal rupture and damage to intestines, bladder, ureters, nerves, or blood vessels. Postoperative complications included any adverse event that occurred within 30 days after surgery, including lymphocysts, thrombosis, infection, and chyle leakage. Visual analog scoring was used to assess the degree of postoperative pain of umbilical incision 24 h after surgery in the range of 0–10, 0 for no pain, 1–3 for mild pain, 4–6 for moderate pain, and 7–10 for severe pain (16). The body image questionnaire (BIQ) was administered 7 and 30 days after the surgery to assess patient satisfaction with the surgical intervention (17). The BIQ consists of two subscales: body image scale and cosmetic scale (Supplementary Data Sheet 1). With a score from 5 to 20, the body image scale measures perception of patients and their attitude to physical condition. The cosmetic scale evaluates the satisfaction of patients to their umbilical scars with a score from 3 to 24. The higher the score, the more satisfied the patient was with body image and cosmetic effect.

### Lymphadenectomy Indications

Dissection of LNs should be recommended for endometrial cancer (EC) patients who are at high risk of recurrence, including those with deep myometrial invasion, high-grade histology, lymphatic vascular invasion, or type II tumors (2). For early-stage EC, the biopsy of sentinel lymph node (SLN) has been proved to be an accurate and effective alternative to lymphadenectomy. However, the use of SLN in high-risk group is controversial, lacking adequate high-level evidences to prove its safety. As a result, systematic lymphadenectomy was nevertheless conducted in this trial on individuals who were considered to be at high risk. In addition, systematic lymphadenectomy was indicated in patients with stage IA-IIA epithelial ovarian carcinoma (OC), except for the mucinous type without suspicious LNs), including those who wished to preserve fertility. Laparoscopy could be employed for patients with early-stage OC by an experienced surgeon (1).

### Surgical Technique

**Figure 3:**
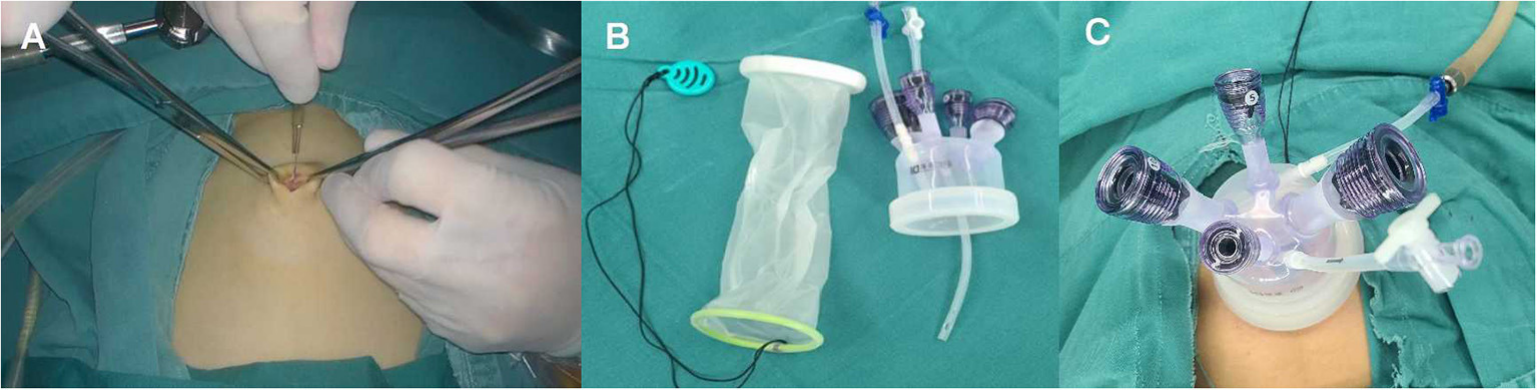
**(A)** Make a 2-cm umbilical incision. **(B)** The multichannel single port (Kangji). **(C)** Set up the port into the intraperitoneal space.

All surgical procedures were performed by an experienced gynecologic oncologist. The patient was placed in trendelenburg position with the primary surgeon on the left and the assistant on the opposite sides. First, the primary surgeon made a 2 cm umbilical incision and a multichannel single port (Kangji, Hangzhou, China) was inserted into the intraperitoneal space (Figure 3). Careful transperitoneal exploration was conducted to exclude intra-abdominal carcinomatosis and collect peritoneal washing for cytologic evaluation.

**Figure 4:**
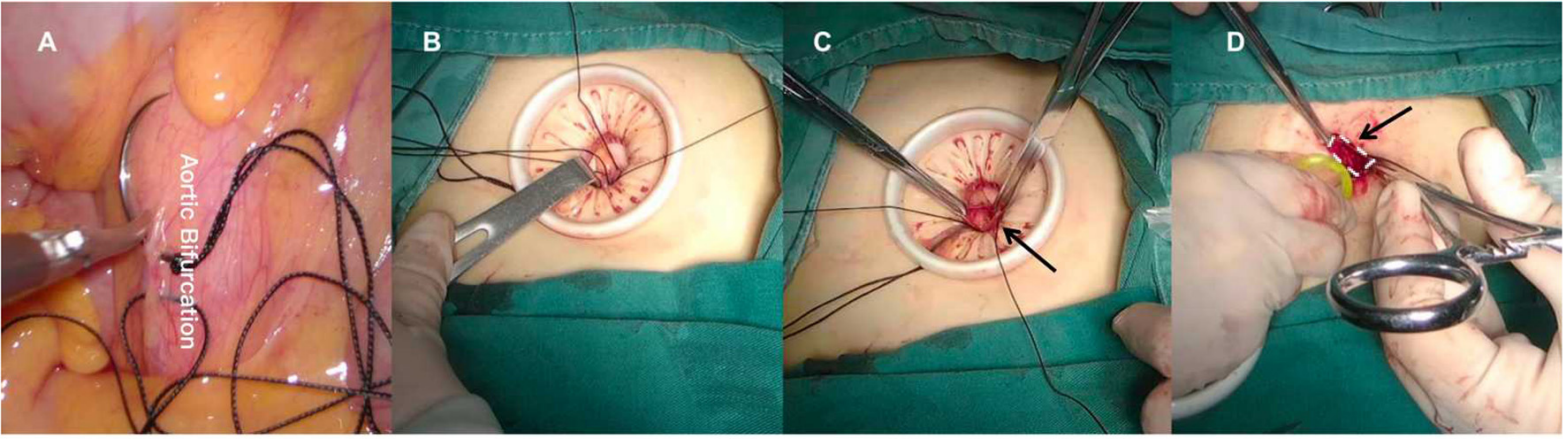
**(A)** Make a figure-of-eight on the posterior peritoneum above the aortic bifurcation. **(B)** Raise the sutured posterior peritoneum to the umbilical incision. **(C)** Cut open the suspended posterior peritoneum. **(D)** Reset the port into the retroperitoneal space.

**Figure 5:**
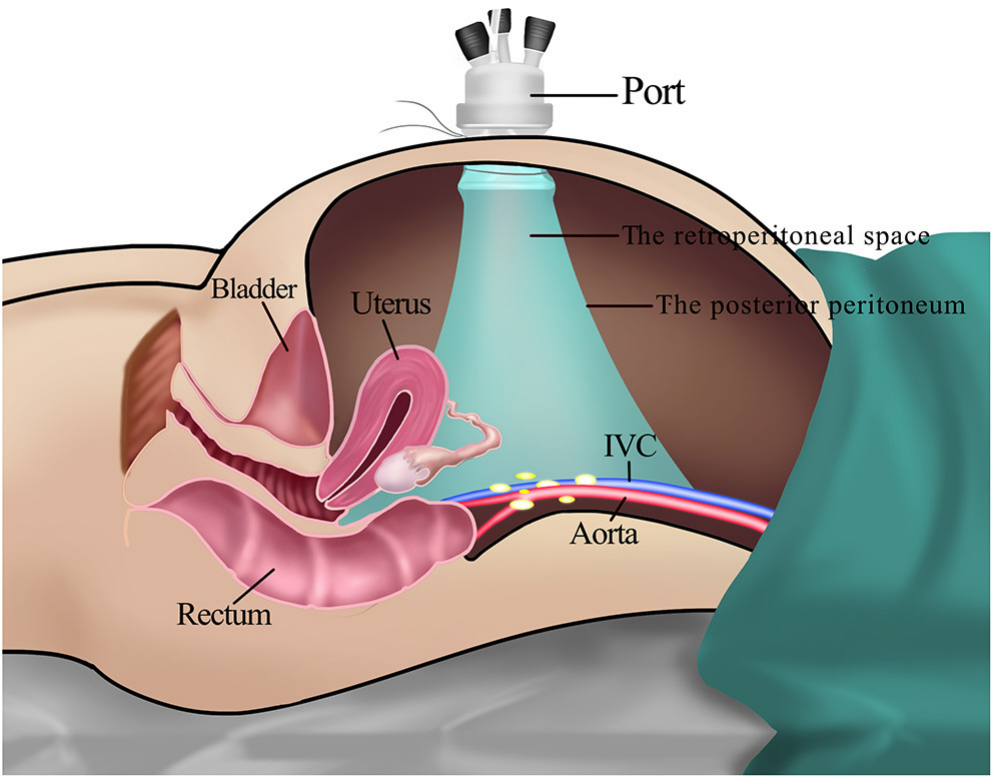
The establishment of the retroperitoneal space. IVC, inferior vena cava.

Second, we had to identify the posterior peritoneum above the aortic bifurcation at first and execute a figure-of-eight suture subsequently. The surgeon pulled the thread and the sutured posterior peritoneum was gently raised toward the umbilical incision. Using a purse-string suture, the suspended posterior peritoneum was held in place and marked. Afterwards, the center portion of the suspended posterior peritoneum was gently sliced open (Figure 4, Supplementary Video 1). The third step was to separate the extraperitoneal soft tissues that attached to the anterior peritoneum with blunt-finger dissection in order to expand the extraperitoneal space. Subsequently, the port was repositioned into the retroperitoneal space with the purse-string suture tightened and secured. The microvessels were coagulated by a harmonic scalpel (HARMONIC, Ethicon, America), and carbon dioxide was insufflated at the maximum pressure of 14–20 mmHg to establish the retropneumoperitoneum (Figure 5, Supplementary Video 1).

**Figure 6:**
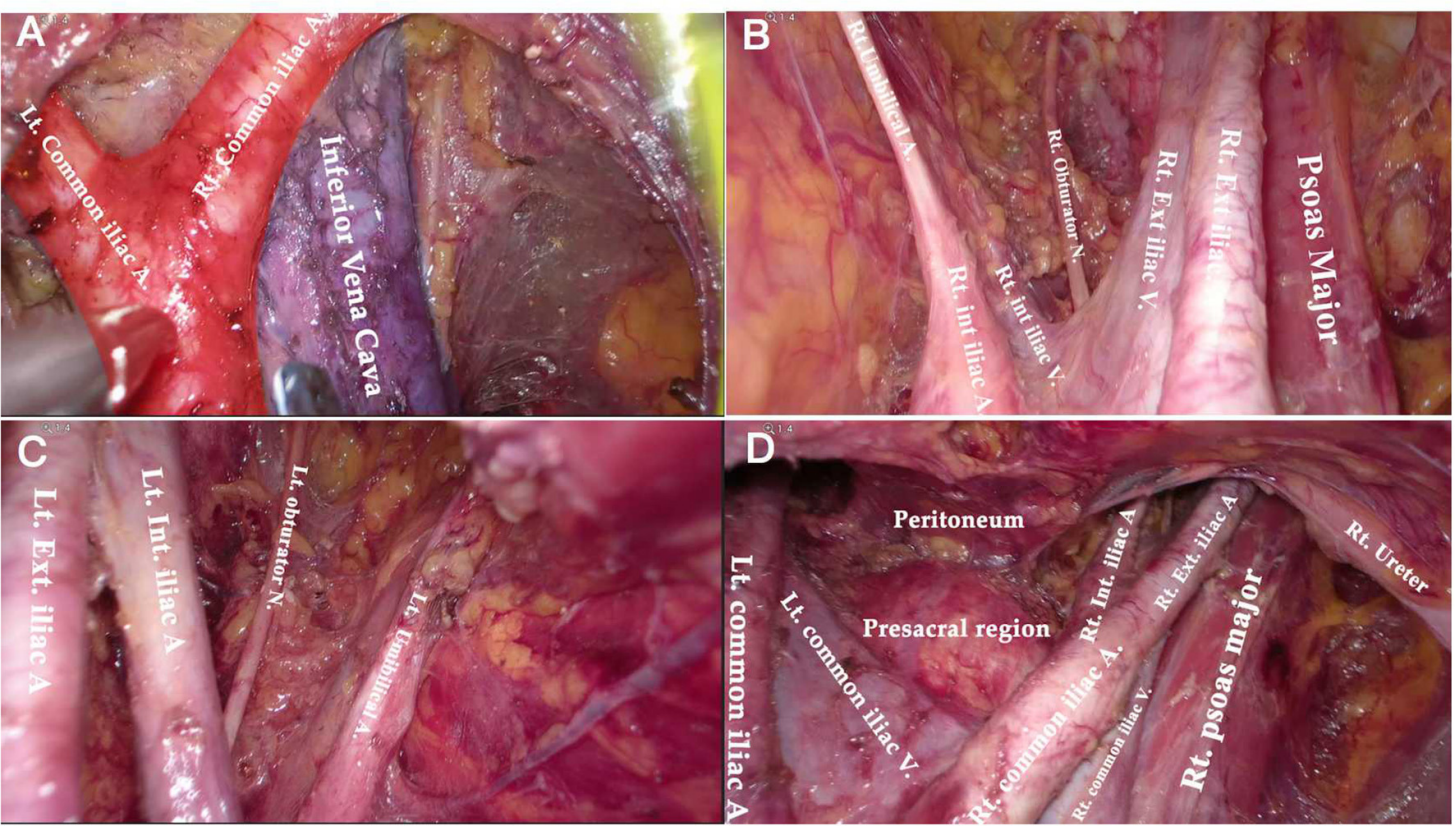
Anatomical overview of pelvic area after PLN. **(A)** The aortic bifurcation and inferior vena cava. **(B)** The right obturator fossa. **(C)** The left obturator fossa. **(D)** The view of presacral area.

**Figure 7:**
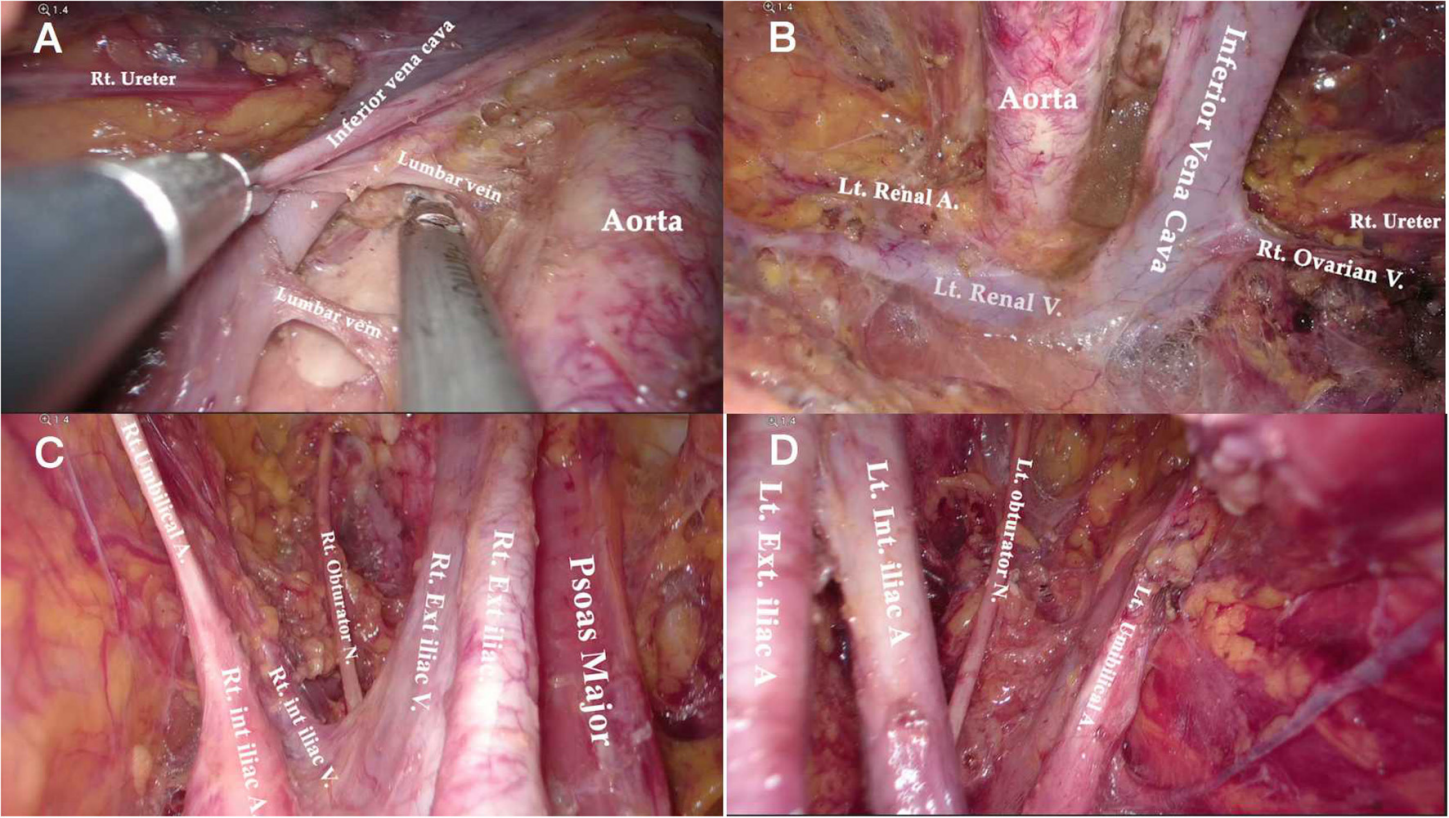
Anatomical overview of para-aortic area. **(A)** Lymphadenectomy up to the left renal vein. **(B)** The right para-aortic region. **(C)** Dissection of the interaortocaval and retrocaval lymph nodes. **(D)** The infrarenal region after PALN.

Procedures were carried out for systematic PLN that included removal of the common iliac, external iliac, internal iliac, obturator, and deep inguinal nodes (Figure 6, Supplementary Video 1). During PALN operations, the surgeon stood between the legs of the patient and the assistant on the right. Para-aortic LNs were dissected from the aortic bifurcation to the left renal vein (RV) (Figure 7, Supplementary Video 1). All surgical specimens were taken out in bags in time to prevent the spillage of tumor cells. And the surgeon sprayed the porcine fibrin sealant kit (Bioseal, Guangzhou, China) onto the surgical field to prevent lymphatic leakage and lymphocyst (18).

After the extraperitoneal surgery, the port was reset into the intraperitoneal space for other transperitoneal procedures (i.e., hysterectomy, omentectomy, or salpinx oophorectomy) according to the different types of tumor.

## Results

### Patient Information

A total of eight patients underwent lymphadenectomy via the TU-LESS extraperitoneal approach. Half of patients were suffering from high-risk EC (one dedifferentiated carcinoma, two grade 3 serous carcinoma with deep myometrial infiltration, and one clear cell carcinoma). The other four patients were diagnosed with early-stage epithelial OC (two serous carcinoma, one clear cell carcinoma, and one endometrioid carcinoma), and three of them opted for fertility-sparing surgery (i.e., preservation of the uterus and contralateral adnexa). The median age was 44 years (range 22–64), and the median BMI was 23.1 kg/m^2^ (range 20.7–28.4). According to the Chinese criteria, two patients were classified as obese (BMI = 28.2 and 28.4 kg/m^2^) (19). In this group, half had a history of abdominal surgery, and one even had undergone four surgeries. The clinical characteristics of patients are summarized in Table 1.

**Table 1:** Patient characteristics.

**Case**	**Age, y**	**BMI, kg/m^**2**^**	**Histologic type**	**FIGO staging**	**Number of previous abdominal surgeries**	**Conversion**
1	48	21.0	Dedifferentiated EC	IIIC	0	N
2	54	23.0	Serous EC	IB	0	N
3	29	28.2	Serous OC	IC	1	N
4	50	26.0	Serous EC	IB	1	N
5	22	22.5	Endometrioid OC	IA	1	N
6	64	28.4	Clear cell EC	IIIC	0	N
7	40	20.7	Serous OC	IA	4	N
8	28	23.1	Clear cell OC	IC	0	N

### Surgical Outcomes

**Figure 8:**
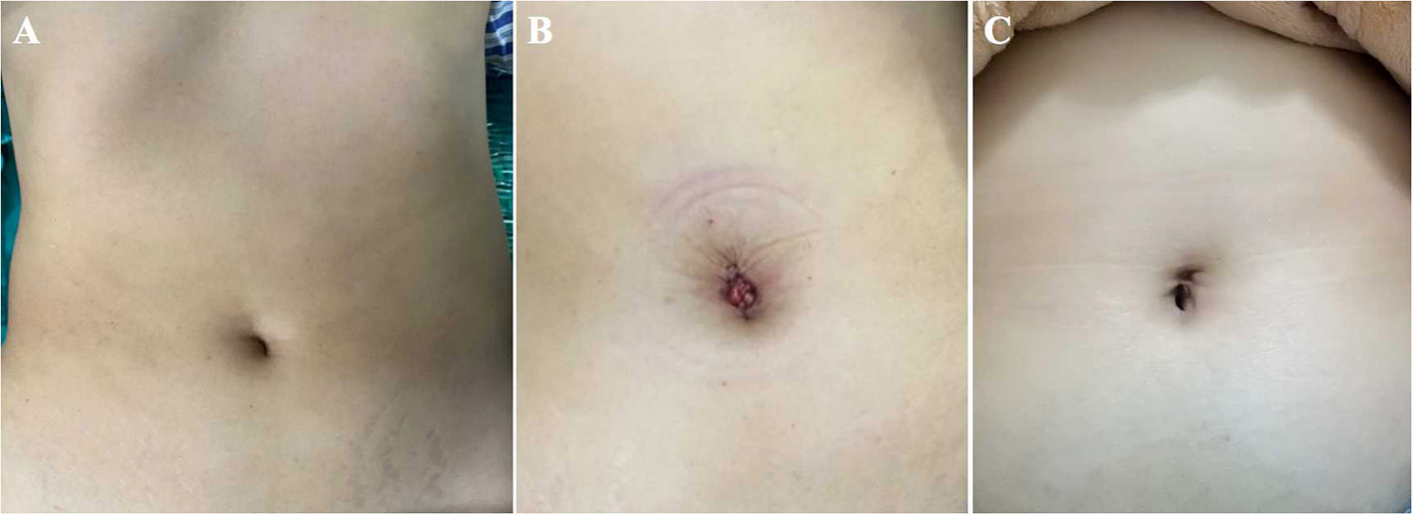
The umbilical incisions of the TU-LESS extraperitoneal approach. **(A)** The preoperative appearance. **(B)** The postoperative appearance. **(C)** The appearance 3 months after surgery.

Table 2 displays the operative outcomes. The upper limit of PALN for all patients was at the renal vascular level. The median time of LN dissection was 166.5 min (range 123–205). During the procedure, no intraoperative complications were observed and no conversion to transperitoneal approach or multiport laparoscopic surgery occurred. The median blood loss was 100 ml (range 100–300) and no patient required blood transfusion. Concerning the LN yields, the median count of para-aortic LNs was 18 (range of 7–30), and the retrieved pelvic LNs was 26.5 (range 18–35). Three EC patients had positive LNs, two with pelvic nodal metastasis and one with para-aortic nodal involvement. Furthermore, there was no evidence of LN metastasis in OC patients. The median flatus time was 23.0 h (range 16.0–38.0) and the median hospital duration was 3 days (range of 2–4). All patients felt mild pain for 24 h after surgery with a median score of 2 (range 1–3). The median satisfaction value for body image was 17 (range 16–19) 7 days after surgery and increased to 19.5 (range 18–20) a month after surgery; while the median score of cosmetic effects was 18 (range 15–19) 1 week after surgery and improved to 22.5 (range 21–23) after 30 days (Figure 8).

**Table 2:** Surgical and postoperative information.

**Case**	**Operative time, min**	**Aortic dissection level**	**Para-aortic LNs, *n***	**Pelvic LNs, *n***	**Blood loss, ml**	**Complications**	**Flatus time, h**	**Postoperative pain score, 24 h**	**Body image scale (range 5–20) 7/30 days**	**Cosmetic scale (range 3–24), 7/30 days**	**Hospital duration, days**
1	205	Infrarenal	14	33	100	N	38	2	16/18	18/22	4
2	173	Infrarenal	43	18	100	N	21	2	17/20	17/23	2
3	163	Infrarenal	12	35	200	N	22	2	18/20	19/23	3
4	165	Infrarenal	19	27	100	N	18	1	19/20	17/23	4
5	175	Infrarenal	30	20	300	N	16	3	16/19	15/22	4
6	168	Infrarenal	17	23	100	N	27	1	16/20	18/22	3
7	158	Infrarenal	21	28	200	N	24	2	17/19	19/21	2
8	123	Infrarenal	7	26	100	N	26	2	18/19	18/23	3
Median	166.5	/	18.0	26.5	100	/	23	2	17/19.5	18/22.5	3

## Discussion

Lymph node status evaluation is a critical component of thorough surgical staging for ovarian and EC (20–22). Laparoscopic lymphadenectomy has been proven safe for surgical staging in EC and early-stage OC patients with less complications and faster recovery (1, 2, 23). Previous studies have shown that extraperitoneal lymphadenectomy is superior to the transperitoneal approach for PALN, because it could avoid intestinal interference and allow an easier access to supramesenteric LNs (5, 24, 25). We initially attempted to employ the TU-LESS extraperitoneal approach in the surgical staging procedures for a patient with advanced cervical cancer in order to accurately delineate the radiographic field. The PALN and right enlarged obturator LN biopsy were performed easily via this novel technique (26). Further exploration and practice of this technique were conducted, and the primary findings of our study confirmed that the TU-LESS extraperitoneal approach is feasible for systematic PLN and PALN.

Compared to the node counts of laparoscopic transperitoneal procedure (range 14–22) (11, 13, 27–29), our method yielded a comparable number of pelvic LNs. The median count of para-aortic LNs (18, range 7–30) in our investigation was equivalent to that of the largest case series of single-port lateral extraperitoneal approach reported by Guoy (median 18, range 2–47) (3), but was higher than that of left-sided extraperitoneal approach using multiport laparoscopy (range 9.5–15) (3, 5, 14, 30). Despite the left extraperitoneal approach being viable for completing the aortic nodal dissection, Dargent asserted that the number of right-sided aortic sampling had reduced compared to bilateral extraperitoneal approach (*p* < 0.01) (31). Furthermore, being limited in access to the deep obturator fossae was one of major technical difficulties of the left-sided approach, which was mainly due to the poor angle of view (13, 15, 32) (as Figure 2C shows). Some technique modifications were made to overcome this difficulty, such as addition of different incisions. Querleu added two incisions on the basis of the left-sided extraperitoneal approach in order to achieve obturator node sampling for patients with locally advanced cervical cancer (32). However, whether this technique could be applied in systematic PLN remains to be verified, and the authors further noted that right obturator fossa was obviously difficult to reach with this technique. Other methods for pelvic LN dissection were also reported, such as combining a right extraperitoneal approach (33). Nonetheless, these modifications would increase surgical trauma, and there were few studies that investigated the feasibility for systematic PLN. One of the greatest merits of the TU-LESS extraperitoneal approach is that it allows equal access to the bilateral pelvic and para-aortic areas through the same extraperitoneal approach because the umbilical incision is centrally located, which facilitates the obturator and infrarenal LN dissection (Figures 6, 7). Additionally, careful contrast of Figures 2C, 6, 7 showed that the anatomic angles during the lateral extraperitoneal procedures were altered, adding to identification complexity for surgeons. However, surgeons did not need to readapt to the changing anatomic angles with the TU-LESS extraperitoneal approach, since the angle of view was the same as the transperitoneal approach or laparotomy which many surgeons have been accustomed to.

Reducing intraperitoneal adhesion is another significant advantage of the extraperitoneal approach. Occelli et al. compared the adhesion rate of laparoscopic transperitoneal vs. extraperitoneal PALN on pigs. The results showed that the extraperitoneal group had a lower adhesion formation rate than the transperitoneal group (*p* = 0.04) (15). Abdominal adhesion is likely to increase the morbidity associated with radiotherapy and may result in adnexal adhesion or even infertility (34, 35). The TU-LESS extraperitoneal approach also theoretically has this advantage, because it could minimize peritoneal injury and leave the peritoneal cavity intact after surgery. For these reasons, in our research, the TU-LESS extraperitoneal approach was considered to be an optimal treatment for young patients who wish to preserve their fertility. Three patients with epithelial OC received the fertility preservation surgery. All of them completed the comprehensive assessment of LNs and dissected lesions with little peritoneal damage. While peritoneal cavity could remain intact with our technique, however, the fertility outcomes ought to be followed. Additionally, patients who underwent abdominal surgeries might potentially benefit from the TU-LESS extraperitoneal approach since it avoids adhesiolysis, and thus it could reduce the risk of intra-abdominal organ injury. In our analysis, half of the patients had surgical history, and none of them had intraperitoneal complications. To sum up, different approaches for lymphadenectomy have their own strengths and limitations; and the concerned summarization from our current exploration and prior studies are presented in Table 3.

**Table 3:** Advantages and limitations of three approaches for lymphadenectomy.

	**Transperitoneal approach**	**Lateral extraperitoneal approach**	**TU-LESS extraperitoneal approach (current work)**
PLN	Easy	Difficult	Easy
PALN(RV level)	Difficult	Easy	Easy
Risk of abdominal adhesion	Increase	Decrease	Decrease
Changes in anatomic recognition	No change	Change	No change
Surgical trauma	Small	Small	Minimal

*RV, renal vein*.

In previous studies, the lymphadenectomy time of the lateral extraperitoneal approach was varied (range 125–339.5 min) (3, 25, 30, 36). The time required for lymphadenectomy in this study was in concordance with the prior findings, but it was less than the time of early practice of the single-port extraperitoneal approach for PALN (average 240 min, range 180–270 min) described by Guoy et al. (37). However, our lymphadenectomy time was somewhat longer than the single-port transperitoneal approach for PLN and PALN (range 60–185 min) (13), which might be explained by the extra time needed to establish the retropneumoperitoneum. The operation time may decrease when the learning curve climbs.

In our research, there were no complications during or after surgery, nor was there a conversion to the transperitoneal route. The procedural failure of the extraperitoneal approach was attributed to the peritoneal rupture (38). Peritoneal rupture occurred in seven patients (16%) during the lateral single-port extraperitoneal lymphadenectomy according to Beytout (14). Neither a peritoneal rupture nor any other technical problems have ever caused abortion of extraperitoneal operation in our series.

Additionally, some studies indicated that the extraperitoneal approach may be an optimal option for patients with a high BMI. Dowdy et al. (39) and Pakish et al. (25) confirmed that patients with BMI >35 kg/m^2^, who had extraperitoneal PALN, harvested more para-aortic nodes than those who underwent abdominal or transperitoneal PALN. BMI had no effect on the duration of surgery, and the area of visceral adipose tissues did not affect the extraperitoneal approach of PALN (40). According to earlier studies, the maximum BMI of patients who underwent the extraperitoneal lymphadenectomy was ranging from 31 to 40 kg/m^2^ (1, 3, 9, 11, 20). Nonetheless, we successfully performed the TU-LESS extraperitoneal technique on two obese patients who satisfied Chinese diagnostic criteria (BMI ≥28 kg/m^2^). However, since this was a primary exploration with a limited number of patients, we did not try to use this measure for systematic lymphadenectomy in patients with BMI more than 30 kg/m^2^. We were exploring an easier method for establishing extraperitoneal space in obese patients. The feasibility and safety of robotic technology for lymphadenectomy in gynecologic cancer have been validated, with the benefits of a three-dimensional vision, scaled movement, and short learning curves (41). Gallotta demonstrated that the robotic technology is conducive for PALN. The results showed that aortic LN yields were comparable when patients with BMI >30 kg/m^2^ were compared with those with BMI <30 kg/m^2^ (42).The robotic surgery was likely to be a preferable approach for obese patients, and the node counts were not affected by increasing BMI (43). Combining robotic technology and the TU-LESS extraperitoneal approach for lymphadenectomy may provide a potential and feasible option for obese patients. Robotic technology may facilitate in shortening the learning curve of the TU- LESS extraperitoneal approach and implementing it.

## Conclusion

In conclusion, the TU-LESS extraperitoneal approach for pelvic and PALN is feasible with a practical application. It significantly improves the exposure and visualization for PLN and PALN, while causing minimal surgical trauma. Depending on the results of our study, this innovative approach may become an effictive alternative measure to the transperitoneal and lateral extraperitoneal approach. However, further studies are required to compare the surgical outcomes like LN yields, surgical trauma, cosmesis, and other index among three approaches. Additionally, based on the current research, a long-term clinical application on a larger sample would be required to evaluate the effects in a more objective manner.

## Data Availability Statement

The original contributions presented in the study are included in the article/Supplementary Material, further inquiries can be directed to the corresponding author.

## Ethics Statement

The studies involving human participants were reviewed and approved by the Medical Institutional Review Board of West China Second Hospital of Sichuan University. The patients/participants provided their written informed consent to participate in this study. Written informed consent was obtained from the individual(s) for the publication of any potentially identifiable images or data included in this article.

## Author Contributions

SP contributed to writing the manuscript and drawing pictures. YZ designed the study and revised the manuscript. FY and KW analyzed and interpreted the data. SC and YW made the video and collected the data. All authors contributed to the manuscript and approved the final manuscript.

## Funding

This work was supported by the Science and Technology Program of Sichuan, China (2020YFS0049), and the Chengdu Science and Technology Bureau (2019-YF05-00473-SN).

## Conflict of Interest

The authors declare that the research was conducted in the absence of any commercial or financial relationships that could be construed as a potential conflict of interest.

## Publisher's Note

All claims expressed in this article are solely those of the authors and do not necessarily represent those of their affiliated organizations, or those of the publisher, the editors and the reviewers. Any product that may be evaluated in this article, or claim that may be made by its manufacturer, is not guaranteed or endorsed by the publisher.

## Supplementary Material

Supplementary Video 1 The TU-LESS extraperitoneal approach for systematic lymphadenectomy.

Supplementary Data Sheet 1 The Body Image Questionnaire (BIQ).
