# Supplementary material for: The Transumbilical Laparoendoscopic Single-Site Extraperitoneal Approach for Pelvic and Para-Aortic Lymphadenectomy: A Technique Note and Feasibility Study
Source: Front Surg. 2022 Apr 15;9:863078. doi: 10.3389/fsurg.2022.863078 (PMC9053588; doi:10.3389/fsurg.2022.863078)
Supplement: Supplementary Data Sheet 1 — The Body Image Questionnaire (BIQ). [file Data_Sheet_1.PDF]

# Body Image Questionnaire（BIQ）

1. Are you less satisfied with your body since the operation?
   Range: 1=no, not at all
   2=a little bit
   3=quite a bit
   4=yes, extremely
2. Do you think the operation has damaged your body?
   Range: 1=no, not at all
   2=a little bit
   3=quite a bit
   4=yes, extremely
3. Do you feel less attractive as a result of your disease or treatment?
   Range: 1=no, not at all
   2=a little bit
   3=quite a bit
   4=yes, extremely
4. Do you feel less feminine/masculine as a results of your disease or treatment?
   Range: 1=no, not at all
   2=a little bit
   3=quite a bit
   4=yes, extremely
5. Is it difficult to look at yourself naked?
   Range: 1=no, not at all
   2=a little bit
   3=quite a bit
   4=yes, extremely
6. On a scale from 1 to 7, how satisfied are you with your (incisional) scar?

| | | | | | | |
|---|---|---|---|---|---|---|
| 1=very unsatisfied | 2 | 3 | 4=not unsatisfied/not satisfied | 5 | 6 | 7= very satisfied |

7. On a scale from 1 to 7, how would you describe your (incisional) scar?

| | | | | | | |
|---|---|---|---|---|---|---|
| 1=revolting | 2 | 3 | 4=not revolting/not beautiful | 5 | 6 | 7= beautiful |

8. Could you score your own incisional scar on a scale from 1 to 10?

*Body image scale: Items 1~5
*Cosmetic scale: Items 6~8
